# Engagement of Nucleotide-binding Oligomerization Domain-containing Protein 1 (NOD1) by Receptor-interacting Protein 2 (RIP2) Is Insufficient for Signal Transduction*

**DOI:** 10.1074/jbc.M114.557900

**Published:** 2014-06-23

**Authors:** Sophie Mayle, Joseph P. Boyle, Eiki Sekine, Birte Zurek, Thomas A. Kufer, Tom P. Monie

**Affiliations:** From the ‡ Department of Biochemistry, University of Cambridge, Cambridge CB2 1GA, United Kingdom,; § Institute for Medical Microbiology, Immunology and Hygiene, University of Cologne, Goldenfelsstrasse 19-21, 50935 Köln, Germany, and; ¶ Department of Veterinary Medicine, University of Cambridge, Cambridge CB3 0ES, United Kingdom

**Keywords:** Caspase, Death Domain, Inflammation, Innate Immunity, NF-κB (NF-κB), Nod-like Receptor (NLR), Protein Kinase, Protein-Protein Interaction, Signal Transduction

## Abstract

Following activation, the cytoplasmic pattern recognition receptor nucleotide-binding oligomerization domain-containing protein 1 (NOD1) interacts with its adaptor protein receptor-interacting protein 2 (RIP2) to propagate immune signaling and initiate a proinflammatory immune response. This interaction is mediated by the caspase recruitment domain (CARD) of both proteins. Polymorphisms in immune proteins can affect receptor function and predispose individuals to specific autoinflammatory disorders. In this report, we show that mutations in helix 2 of the CARD of NOD1 disrupted receptor function but did not interfere with RIP2 interaction. In particular, N43S, a rare polymorphism, resulted in receptor dysfunction despite retaining normal cellular localization, protein folding, and an ability to interact with RIP2. Mutation of Asn-43 resulted in an increased tendency to form dimers, which we propose is the source of this dysfunction. We also demonstrate that mutation of Lys-443 and Tyr-474 in RIP2 disrupted the interaction with NOD1. Mapping the key residues involved in the interaction between NOD1 and RIP2 to the known structures of CARD complexes revealed the likely involvement of both type I and type III interfaces in the NOD1·RIP2 complex. Overall we demonstrate that the NOD1-RIP2 signaling axis is more complex than previously assumed, that simple engagement of RIP2 is insufficient to mediate signaling, and that the interaction between NOD1 and RIP2 constitutes multiple CARD-CARD interfaces.

## Teaser

**Background:** Protein/protein interactions are critical for signal transduction.

**Results:** Nucleotide-binding oligomerization domain-containing protein 1 (NOD1) helix 2 mutants impair signaling but not interaction with receptor-interacting protein 2 (RIP2).

**Conclusion:** NOD1 and RIP2 interaction is necessary, but not sufficient, for NOD1 signaling.

**Significance:** NOD1 signaling is more complex than previously assumed and is likely to involve multiple CARD interfaces.

## Introduction

Nucleotide-binding leucine-rich repeat-containing (NLR)^3^ proteins are key components of the innate immune system. Many NLRs serve as cytoplasmic sentinels responding to both exogenous and endogenous danger signals. NLRs have a tripartite domain organization with an N-terminal effector domain required for signal transduction, a central nucleotide binding domain reportedly involved in protein oligomerization, and a C-terminal leucine-rich repeat domain essential for ligand detection and receptor stimulation (1–4). Activation of NLR family members can result in a proinflammatory immune response and/or formation of an inflammasome and subsequent activation of caspase-1 (1).

The prototype NLR proteins, NOD1 and NOD2, are activated by the bacterial peptidoglycan fragments d-Glu-*meso*-diaminopimelic acid (iE-DAP) and muramyl dipeptide, respectively. Activation of NOD1/2 results in receptor translocation from the cytoplasm to the plasma membrane and precipitates homotypic interactions between the effector CARDs of the receptors and their adaptor protein RIP2 (also known as RICK) (5–8). RIP2 is subsequently autophosphorylated at Tyr-474, precipitating a series of ubiquitination events involving ubiquitin ligases, such as X-linked inhibitor of apoptosis and the cellular inhibitor of apoptosis proteins 1 and 2 (9–12). Defects in NOD2, such as the Crohn disease-associated mutation L1007fsincC, result in a loss of RIP2 autophosphorylation and a lack of response to ligand stimulation (9). Signal transduction via RIP2 leads to induction of the NFκB pathway (13, 14). NOD2 has also been shown to signal via CARD9 to activate the p38 and c-Jun N-terminal kinase mitogen-activated protein kinase pathways (15), to activate the non-canonical NFκB pathway via engagement of NFκB-inducing kinase (16), and to activate interferon-regulatory factor 3 following recognition of viral single-stranded RNA (17).

The biological contribution of NOD1 and NOD2 to the mammalian innate immune response has been well documented. However, the precise structural and molecular details of NOD1 and NOD2 activation remain largely elusive. Interactions between the effector domains of NOD1 and RIP2 have been studied by a number of groups (13, 14, 18, 19). These studies have shown that an acidic patch on helix 3 of the NOD1 CARD forms the primary binding interface with basic residues in RIP2 predicted to be located on the first and fourth helices (18).

In this study, we used a combination of functional and biochemical studies to thoroughly investigate the precise nature of the interaction between the CARDs of NOD1 and RIP2. Mutations in regions of helix 2 of the NOD1 CARD interfered with signal transduction but did not affect interaction with RIP2 or the cellular localization of NOD1. Studies with recombinant proteins suggest that this functional impact may be the result of alterations in the propensity of the NOD1 CARD to self-associate. Mutational analysis of the RIP2 CARD suggested that Lys-443 and Tyr-474, a previously described site of autophosphorylation, may also be important for permitting interaction with NOD1. Comparative analysis with the structures of known CARD-CARD interfaces indicates that the interaction between NOD1 and RIP2 is likely to involve type I and type III, but not type II, interfaces, thereby suggesting that formation of the downstream signaling complex may be more complex than previously assumed.

## EXPERIMENTAL PROCEDURES

### 

#### 

##### Plasmids

pUNO-NOD1, encoding full-length untagged NOD1, was a kind gift from Dr. P. Murray; pCMV-NOD1 produces full-length NOD1 with an N-terminal FLAG tag (20); pCI-RIP2-Myc-His, pEF6-CARD9-V5, and GB1-RIP2-CARD were kind gifts from Drs. K. Fitzgerald, D. Underhill, and K. Rittinger, respectively; pLuc and phrG (Promega) encode firefly and *Renilla* luciferase, respectively. The NOD1 CARD domain (residues 15–110) was cloned into pDEST-HisMBP (21), pGEX4T1, and pET28 vectors to enable expression of His-maltose-binding protein (MBP), glutathione *S*-transferase (GST), and His_6_-tagged protein. Site-directed mutagenesis was used to generate mutant NOD1 constructs in pUNO, pCMV, pET28, and pGEX4T1 backbones and mutant GB1-RIP2-CARD constructs.

##### Luciferase Reporter and Co-immunoprecipitation Assays

HEK293 cells were maintained in DMEM (Sigma) supplemented with 10% FCS, 100 μg/ml penicillin/streptomycin, and 2 mm l-glutamine at 37 °C and 5% CO_2_. Cells were transfected using jetPEI (Polyplus Transfection) with 0.1 ng/well NOD1 DNA and 1 ng/well pLuc and phrG in a 96-well plate. Cells were stimulated with specified concentrations of either iE-DAP (Invivogen) or the control ligand iE-Lys (Invivogen) concomitantly with DNA transfection. Cells were lysed 24 h post-transfection with 1× passive lysis buffer (Promega), and luminescence measured with a LUMIstar Luminometer (BMG Labtech). Protein expression was checked 24 h after transfection of HEK293 cells with 3 μg of DNA/well in a 6-well plate without ligand stimulation. NOD1 was visualized by Western blot using the NOD1 monoclonal antibody 2A10 (6). For co-immunoprecipitations, HEK293 cells were transfected with 1.5 μg of both pCMV-NOD1 and pCI-RIP2-MycHis in the presence and absence of 100 ng/ml iE-DAP. After 24 h, cells were washed twice in 1× PBS and lysed in 400 μl of radioimmune precipitation assay buffer (50 mm Tris-HCl, pH 7.6, 150 mm NaCl, 0.25% Triton X-100, 0.1% SDS, 0.5% sodium deoxycholate) supplemented with 1× Protease Inhibitor Mixture set V (Calbiochem) and 125 units of Benzonase nuclease (Sigma)/well. Lysates were incubated on ice for 10 min with shaking and clarified by centrifugation (16,000 × *g*; 2 min; 20 °C). Supernatants were incubated with anti-FLAG antibody (Sigma; F3165) immobilized on Protein G-coated magnetic Dynabeads® (Invitrogen) according to the manufacturer's protocol. Beads were washed, and immunoprecipitated proteins were eluted under denaturing conditions before detection by Western blotting with the specified antibodies.

##### Immunofluorescence

HeLa cells were seeded on glass coverslips and transiently transfected with 1 μg of the expression plasmids using FuGENE 6 transfection reagent (Roche Applied Science) according to the manufacturer's instructions. After 24 h, cells were fixed with 3% paraformaldehyde (Roth) in PBS for 10 min and permeabilized with 0.5% Triton X-100 (Roth) in cold PBS for 5 min. Cells were blocked in 3% BSA (Roth) in PBS for 20 min and incubated successively in mouse anti-FLAG M2 (1:20,000; Stratagene) and goat anti-mouse Alexa Fluor 546 (1: 200; Invitrogen Molecular Probes) antibodies. DNA was stained with DAPI (5 μg/ml; Invitrogen Molecular Probes), and actin was stained with phalloidin-FITC (2.5 μg/ml; Sigma-Aldrich). Cells were mounted in ProLong Gold antifade reagent (Invitrogen Molecular Probes). Image acquisition of z-stacks was performed on an Olympus FV-1000 laser-scanning microscope (objective, Olympus PlanApo, 60×/1.40 oil, 8/0.17) and processed using ImageJ software.

##### Subcellular Fractionation

Membrane and cytosolic fractionation of transfected HEK293 cells was performed using a Subcellular Fractionation kit (Pierce) according to the manufacturer's instructions. Antibodies against Flotillin-2 (Abcam) and GAPDH (Abcam) were used to characterize the membrane and cytosolic fractions.

##### Expression of His-tagged Recombinant NOD1 CARD

Proteins were expressed from pET28-NOD1 CARD(15–110) or the respective mutant in Terrific broth overnight at 20 °C. Bacterial pellets were stored at −20 °C, resuspended in lysis buffer (25 mm sodium phosphate, pH 7.3, 300 mm NaCl, 20 mm imidazole, 0.1% Triton X-100 (v/v), 5 mm β-mercaptoethanol, 1 mg/ml lysozyme, 250 units of Benzonase (Sigma), 1× Protease Inhibitor Mixture set V), and lysed by sonication, and recombinant protein was recovered using nickel-nitrilotriacetic acid affinity chromatography and size exclusion chromatography (25 mm sodium phosphate, 100 mm NaCl, ±2 mm DTT; Superdex 75 HiLoad 16/60 column, GE Healthcare).

##### GST Co-purification and Pulldown Assays

GST-NOD1-CARD or His-MBP-NOD1-CARD and GB1-RIP2-CARD were expressed in separate 50-ml Luria broth cultures overnight at 20 °C using 1 mm isopropyl 1-thio-β-d-galactopyranoside. Cells were harvested (8,000 × *g*, 4 °C, 15 min), and bacterial pellets were stored at −20 °C before resuspension in 0.5 ml of lysis buffer supplemented with 50 units of Benzonase nuclease (Sigma), 1 mg/ml lysozyme, and 1× Protease Inhibitor Mixture set V. Lysates were incubated with rotation for 5 min at room temperature and then mixed as appropriate. Samples were sonicated, clarified (4 °C, 16,000 × *g*, 10 min), incubated with 150 μl of glutathione-Sepharose 4B (GE Healthcare) (pre-equilibrated in lysis buffer) for 30 min at 25 rpm at 4 °C, washed with 15 ml of lysis buffer, eluted in 300 μl of elution buffer (50 mm Tris, pH 8.0, 100 mm NaCl, 1 mm DTT, 50 mm reduced glutathione), and analyzed by SDS-PAGE.

##### Circular Dichroism

Proteins were buffer-exchanged into 25 mm sodium phosphate, pH 7, 100 mm sodium fluoride, 1 mm tris(2-carboxyethyl)phosphine (TCEP) and centrifuged (16,000 × *g*, 10 min, 4 °C); and 400 μl was loaded into a 1.0-mm quartz cuvette. CD scans were performed using an Aviv model 4.0 CD spectrophotometer. Machine units were converted to mean residue ellipticity, [θ], using Dichroweb (22) and analyzed with GraphPad Prism 4. Secondary structure composition was assessed using SELCON3 via Dichroweb.

##### Analytical Ultracentrifugation

Sedimentation velocity experiments were performed in a Beckman Optima XL-I analytical ultracentrifuge equilibrated at 20 °C with 400 μl of 2 mg/ml protein in 25 mm sodium phosphate, 100 mm NaCl at pH 7 in the presence and absence of 1 mm TCEP. Proteins were spun at 50,000 rpm (201,500 × *g*), and 200 scans were collected using interference optics. Scans were analyzed and refined using SEDFIT (23), and data are presented with GraphPad Prism 4.

##### Dynamic Light Scattering

To analyze the size distribution of particles within purified protein solutions, dynamic light scattering was performed using a Malvern Zetasizer Nano S DLS machine. Protein samples were buffer-exchanged into 25 mm sodium phosphate, pH 7, 100 mm NaCl, 5 mm β-mercaptoethanol, and contaminants were removed by centrifugation (16,000 × *g*, 10 min, 4 °C). The experiment was performed at ∼21 °C, and three sets of scans were performed per sample. Experimental data were analyzed using the Dispersion Technology Software 5.02 (Malvern Instruments).

##### Homology Modeling and Bioinformatics

Amino acids corresponding to the RIP2 CARD (amino acids 435–528) were submitted to the homology search server pGENThreader (24). The top hit was the NOD1 CARD (Protein Data Bank code 2DBD; score, 44.083; *p* value, 0.002), which was used as a template for model building with Modeler version 9.8 (25). Side-chain conformations were optimized using SCWRL4 (26) before refinement using Modeler. Stereochemistry was analyzed using MolProbity (27), which indicated excellent stereochemistry for the RIP2 CARD model. 97.6% of residues were found in the favored regions of the Ramachandran plot; there were no outliers, and no residues possessed poor rotamers.

Full-length human NOD1 (GenBank accession number NP_006083.1) was used to perform a BLASTp (28) search of the non-redundant protein database, and 31 full-length NOD1 orthologues were collated and aligned using ClustalW2 (29). The potential impact of NOD1 CARD mutants was predicted using PolyPhen-2 (30) using the classifier model HumDiv and genome assembly GRCh37/hg19. The interface between subunits in the NOD1 crystal dimer structure was analyzed using PDBePISA (31). SNP frequencies were retrieved from the Exome Variant Server.

## RESULTS

### 

#### 

##### Revisiting the Nature of the Interface between NOD1 and RIP2

**FIGURE 1.**
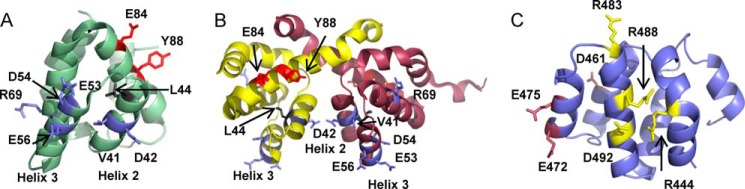
**Key functional residues in the CARD of NOD1 and RIP2.** Previously identified functionally important amino acids were mapped in *stick* representation onto the monomeric (*A*) and dimeric (*B*) forms of the NOD1 CARD (Protein Data Bank codes 2DBD and 2NZ7, respectively) and a model of the RIP2 CARD (*C*). For the NOD1 CARDs, mutation of residues colored *blue* (Val-41, Asp-42, Glu-53, Asp-54, Glu-56, and Arg-69) has been shown to inhibit both receptor signaling and RIP2 binding; for residues colored *gray* (Leu-44) signaling was impaired, but RIP2 binding was unaffected; and residues colored *orange* (Glu-84 and Tyr-88) have been implicated in ubiquitin binding, and their mutation enhances signaling. Helices 2 and 3 in NOD1 are labeled. In RIP2, the residues are split into two potential interfaces containing Arg-444, Arg-483, Arg-488, and Asp-492 (*yellow*) and Asp-461, Glu-472, and Glu-475 (*orange*).

Protein/protein interactions and the assembly of macromolecular signaling platforms are essential for innate immune signal transduction. For NOD1, a key interaction is between its CARD and the CARD of the adaptor protein RIP2. Previously, three acidic residues (Glu-53, Asp-54, and Glu-56) in helix 3 of the NOD1 CARD and three basic residues (Arg-444, Arg-483, and Arg-488) in the RIP2 CARD were identified as key mediators of the NOD1-RIP2 interaction (Fig. 1*A*-C) (18). This study also showed that double and triple mutants in helix 2 of the NOD1 CARD abrogated signaling and seriously impaired interaction with RIP2. Recently, we have demonstrated that NOD1 Glu-56 may in fact not be critical for this interaction (32). Meanwhile, Fridh and Rittinger (33) have shown that the NOD2-RIP2 interface may differ from that between NOD1 and RIP2 and could involve basic residues on NOD2 and acidic residues on RIP2; mutation of a number of these acidic RIP2 residues also stopped interaction with NOD1. Other recent studies indicate that CARD-CARD interactions involve multiple interfaces and may have a complexity comparable with that of death domains (34–36). In light of this, we decided to take a closer look at the nature of the interaction between NOD1 and RIP2.

We began by analyzing the distribution of residues implicated in NOD1 and RIP2 CARD-CARD interaction in the context of the three types of interface (Table 1) defined for members of the death domain superfamily in the structure of the PIDDosome (p53-induced death domain protein) (37). Except for Arg-69, which is found in helix 4, NOD1 residues implicated as important for RIP2 interaction mapped to helices 2 and 3 (Table 2 and Fig. 1, *A* and *B*). As the structure of the RIP2 CARD has not yet been determined, we generated a homology model to approximate the position of the helices. Residues in RIP2 identified by Manon *et al.* (18) and Fridh and Rittinger (33) as important for NOD1 interaction are dispersed across helices 1, 2, 3, and 4 but cluster into two distinct surfaces (Fig. 1*C*).

**TABLE 1:**
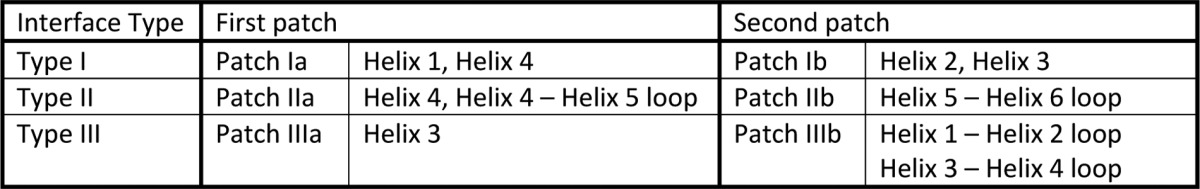
**Regions contributing to death domain family member interaction interfaces**

**TABLE 2:** **NOD1 residues involved in dimerization and signal transduction** ND, not determined. Dimer interface data were obtained from PDBePISA analysis. Functional data were obtained from the sources indicated.

NOD1 residue	Structural location	Contribution to dimer interface		Impact on NOD1 function
		Chain A	Chain B	
		Å*^2^*		
Leu-22	Helix 1	15.37	15.18	ND
Leu-23	Helix 1	0.50	0.00	ND
Asn-26	Helix 1	21.80	19.03	ND
Leu-29	Helix 1	37.82	37.12	ND
Leu-30	Helix 1	19.47	22.25	ND
His-33	Helix 1	57.02	66.53	ND
Ile-34	Helix 1	20.71	15.90	ND
Arg-35	Helix 1-helix 2 loop	86.68	99.79	ND
Asn-36	Helix 1-helix 2 loop	60.34	48.55	ND
Gln-38	Helix 2	50.24	55.41	ND
Cys-39	Helix 2	61.48	53.99	ND
Leu-40*^a^*	Helix 2	3.79	1.17	None
Val-41*^a^*	Helix 2	0.00	0.00	V41A and V41Q both slightly reduce signaling, and V41A has reduced RIP2 binding
Asp-42*^a^*	Helix 2	35.51	36.16	Inhibits signaling as double/triple mutant
Asn-43	Helix 2	20.64	17.32	ND
Leu-44*^a^*	Helix 2	0.00	0.00	Extensive inhibition of signaling; normal RIP2 binding
Lys-46	Helix 2	32.72	30.73	ND
Asp-48*^a^*	Helix 2-helix 3 loop	0.00	0.00	None
Ala-52*^a^*	Helix 3	0.00	0.00	None
Glu-53*^a^*^,^*^b^*	Helix 3	0.00	0.00	Abrogates signaling and RIP2 interaction
Asp-54*^a^*^,^*^b^*	Helix 3	0.00	0.00	Abrogates signaling and RIP2 interaction
Glu-56*^a^*^,^*^b^*	Helix 3	0.00	0.00	E56K abrogates signaling and RIP2 interaction; E56A does not
Ile-57*^a^*	Helix 3	0.00	0.00	None
Lys-67*^a^*	Helix 4	0.00	0.00	None
Arg-69*^a^*^,^*^c^*	Helix 4	0.00	0.00	Abrogates signaling; significant reduction in RIP2 binding
Glu-84*^c^*	Helix 5	0.00	0.00	Implicated in ubiquitin binding; enhanced signaling on mutation
Leu-87	Helix 5	24.03	22.95	ND
Tyr-88*^c^*	Helix 5	0.00	0.00	Implicated in ubiquitin binding; enhanced signaling on mutation
Leu-90	Helix 5	37.02	34.63	ND
Gln-91	Helix 5	34.71	37.96	ND
Gln-92	Helix 5	0.17	7.20	ND
Leu-93	Helix 5	48.62	55.02	ND
Ala-94	Helix 5	64.12	66.23	ND
Asp-95	Helix 5	48.40	51.00	ND
Ala-96	Helix 5	54.95	55.68	ND
Tyr-97	Helix 5	141.34	141.41	ND
Val-98	Helix 5-helix 6 loop	38.07	35.90	ND
Asp-99	Helix 5-helix 6 loop	59.20	69.47	ND
Leu-100	Helix 6	79.12	87.33	ND
Arg-101	Helix 6	82.71	71.36	ND
Trp-103	Helix 6	102.87	103.49	ND
Leu-104	Helix 6	82.34	85.83	ND
Leu-105	Helix 6	0.68	0.00	ND

*^a^* Manon *et al.* (18).

*^b^* Boyle *et al.* (32).

*^c^* Ver Heul *et al.* (19).

**FIGURE 2.**
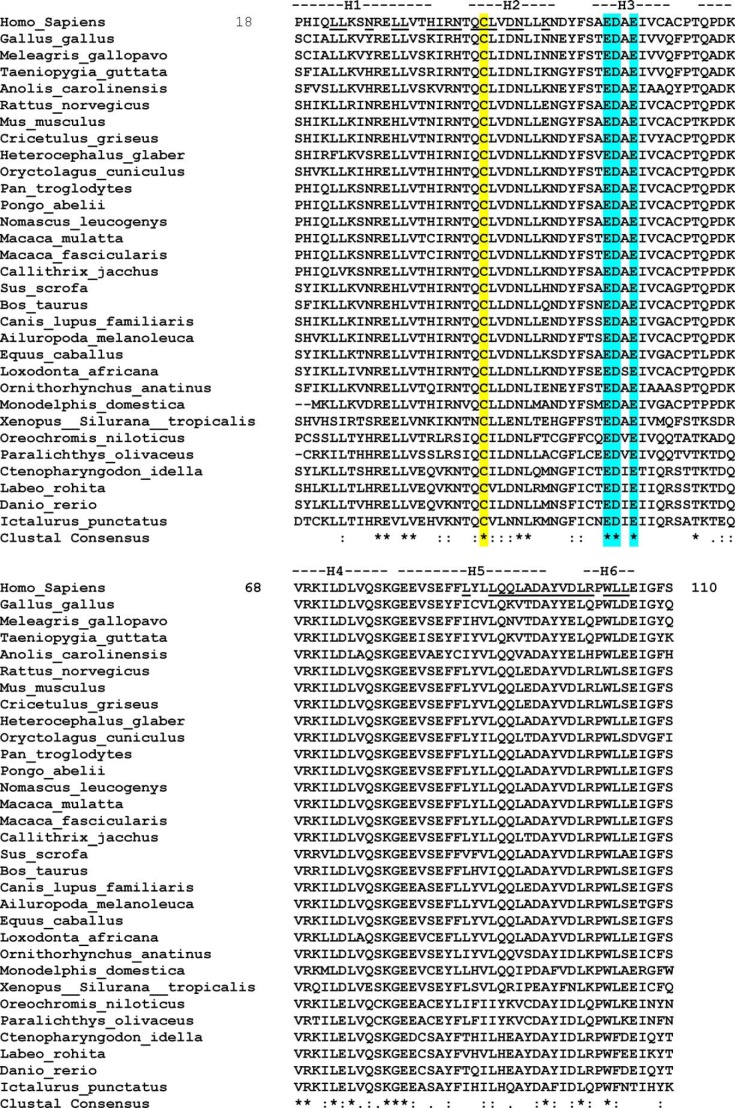
**ClustalW2 multiple sequence alignment of the NOD1 CARD from 31 different species.** For clarity, human NOD1 has been placed at the *top* of the alignment. The conserved Cys-39 residue and the core RIP2 binding motif are highlighted in *yellow* and *cyan*, respectively, across all species, whereas residues involved in the dimer interface of the NOD1 CARD crystal structure are *underlined* in the human sequence. In the consensus sequence, complete conservation is denoted by an *asterisk* (*), strongly conservative substitutions are denoted by a *colon* (:), and partially conservative substitutions are denoted by a *dot* (.). GenBank accession numbers for the sequences are as follows: *Gallus gallus*, XP_418777.2; *Meleagris gallopavo*, XP_003207269.1; *Taeniopygia guttata*, XP_002196320.1; *Anolis carolinensis*, XP_003222248.1; *Rattus norvegicus*, NP_001102706.1; *Mus musculus*, NP_766317.1; *Cricetulus griseus*, XP_003507840.1; *Heterocephalus glaber*, EHB11938.1; *Oryctolagus cuniculus*, XP_002713781.1; *Homo sapiens*, NP_006083.1; *Pan troglodytes*, XP_001165528.1; *Pongo abeli*, XP_002818130.1; *Nomascus leucogenys*, XP_003270528.1; *Macaca mulatta*, EHH17407.1; *Macaca fascicularis*, EHH52238.1; *Callithrix jacchus*, XP_002751479.1; *Sus scrofa*, NP_001107749.1; *Bos taurus*, XP_598513.3; *Canis lupus familiaris*, XP_539499.2; *Ailuropoda melanoleuca*, XP_002919315.1; *Equus caballus*, XP_001499616.1; *Loxodonta africana*, XP_003407068.1; *Ornithorhynchus anatinus*, XP_001512159.1; *Monodelphis domestica*, XP_001381520.2; *Xenopus* (*Silurana*) *tropicalis*, XP_002937900.1; *Oreochromis niloticus*, XP_003446247.1; *Paralichthys olivaceus*, AFD29894.1; *Ctenopharyngodon idella*, ACX71752.1; *Labeo rohita*, AFE61355.1; *Danio rerio*, XP_002665106.2; and *Ictalurus punctatus*, NP_001186996.1. *H1–H6*, helices 1–6.

In its crystalline form, the NOD1 CARD forms a dimer due to helix swapping between helices 1 and 6 (Fig. 1*B*) (38, 39). Further stabilization is provided by an interprotomer disulfide bond mediated by Cys-39. This residue is conserved across NOD1 species (Fig. 2). We used PDBePISA to determine the contribution of residues in NOD1 to the dimer interface (Table 2 and Fig. 1*B*). Residues across helices 1, 2, 5, and 6 and the connecting loops contributed to the dimer interface. Only helix 2 is currently implicated in both NOD1 dimerization and signal transduction (Table 2).

##### Mutation of NOD1 Helix 2 Results in Impaired Activation of NFκB by NOD1

**FIGURE 3.**
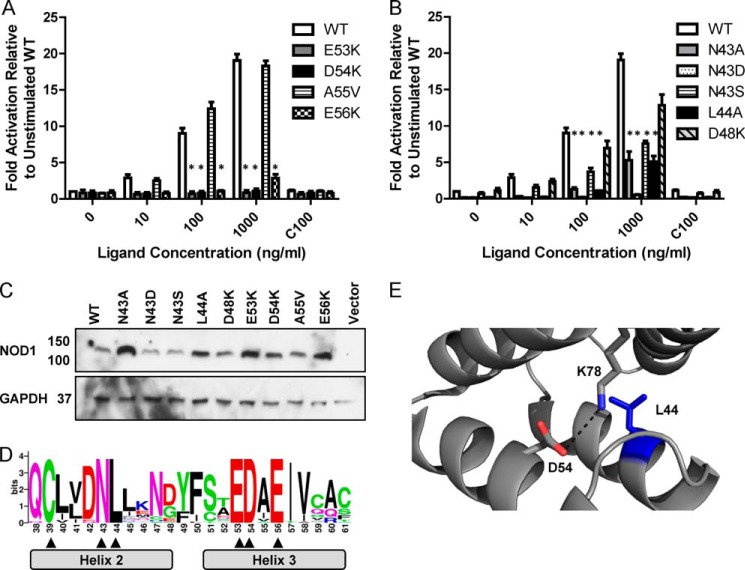
**Mutation of Asn-43 results in NOD1 dysfunction in response to ligand stimulation.** NFκB luciferase reporter assays were performed in HEK293 cells using wild-type NOD1 (*WT*) and receptors mutated in helix 3 (*A*) or helix 2 (*B*). DNA (0.1 ng/well) and the indicated concentration of stimulatory (iE-DAP) or 100 ng/ml of the control ligand ie-Lys (C100) were transfected into 96-well plates. After 24 h, cells were lysed, and NFκB activity was determined. Results show the average of four independent experiments, and * represents *p* < 0.0005 compared with wild type. *Error bars* indicate S.E. *C*, immunoblots of expression levels of NOD1 WT and mutant constructs. Immunoblot samples (3 μg of DNA/well in a 6-well plate) were lysed after 24 h and probed with the indicated antibodies. Immunoblots are representative of at least three separate experiments. *D*, WebLogo representation of NOD1 CARD helices 2 and 3, the core region of NOD1 implicated in signaling and RIP2 interaction. Residue numbering matches the human sequence. Residue *height* represents the degree of conservation across 31 full-length NOD1 sequences from different mammalian species. Absolutely conserved residues are indicated with an *arrowhead. E*, Leu-44 (*blue*) is an internal hydrophobic residue, and Asp-54 forms an internal salt bridge with Lys-78. The salt bridge is denoted by a *dashed black line* and has a distance of 3.8 Å.

HEK293 cell-based NFκB reporter assays were used to assess the signaling capabilities of wild-type NOD1 and various CARD mutants across helices 2 and 3. Mutants were generated by site-directed mutagenesis in pUNO-hNOD1, transiently transfected into HEK293 cells, and stimulated with increasing doses of the NOD1 agonist iE-DAP or a constant dose of an inactive control compound, iE-Lys. Consistent with previous work (18), the helix 3 mutants E53K, D54K, and E56K all abrogated NOD1 signaling (Fig. 3*A*). The other helix 3 mutant tested, A55V (which exists as a polymorphism in 1 in 2,000 African-Americans), activated NFκB at a level at least comparable with the wild-type protein.

Double and triple mutants in helix 2 of the NOD1 CARD have been shown to disrupt signaling and interaction with RIP2 possibly due to structural perturbation (18). However, with the exception of L44A, single mutants have not been shown to disrupt NOD1-mediated NFκB signaling. We confirmed the impact of L44A on NOD1 signaling (Fig. 3*B*). We also tested the impact of mutating the surface-exposed residue Asn-43. This residue was mutated to an alanine, a serine (a reported polymorphism in the NCBI database), and an aspartic acid (to maintain size but change residue properties from polar to acidic). We found that N43D failed to respond to any ligand stimulation, and both N43A and N43S were significantly impaired in their response at ligand concentrations of 100 and 1000 ng/ml (Fig. 3*B*). Interestingly, N43A, N43D, and L44A showed a significant reduction in basal signaling (Fig. 3*B*). When compared with these basal levels, the fold activation following ligand stimulation of N43A and L44A, but not N43D, was similar to the change in wild-type NOD1 albeit with a somewhat lower level of signaling response. This was not the result of dramatic changes in receptor expression level (Fig. 3*C*). Together the various changes in basal and ligand-induced signaling suggest an important role for helix 2 in NOD1 signaling. None of the mutant constructs behaved as dominant negatives when tested (data not shown).

The functional importance of the residues identified in helices 2 and 3 for NOD1 function is highlighted by their evolutionary conservation (Figs. 2 and 3*D*) (32). Interestingly, Cys-39, Asn-43, Leu-44, Glu-53, Asp-54, and Glu-56 were the only residues in this region showing absolute conservation. Of these, Cys-39 has already been shown to be important in NOD1 CARD dimerization, whereas Glu-53, Asp-54, and Glu-56 are all crucial for RIP2 interaction and signal transduction. On evaluation of the tertiary structure of NOD1, it is clear that Leu-44 forms part of the hydrophobic core (Figs. 1*A* and 3*E*) and that Asp-54 is actually involved in an internal salt bridge with Lys-78 (Fig. 3*E*), hence explaining why mutation of these residues impairs NOD1 function. Consequently, the complete cross-species conservation of Asn-43 is strong support for a crucial role of this residue in NOD1 function.

##### Mutation of Residues in NOD1 Helices 2 and 3 Does Not Affect Membrane Recruitment

**FIGURE 4.**
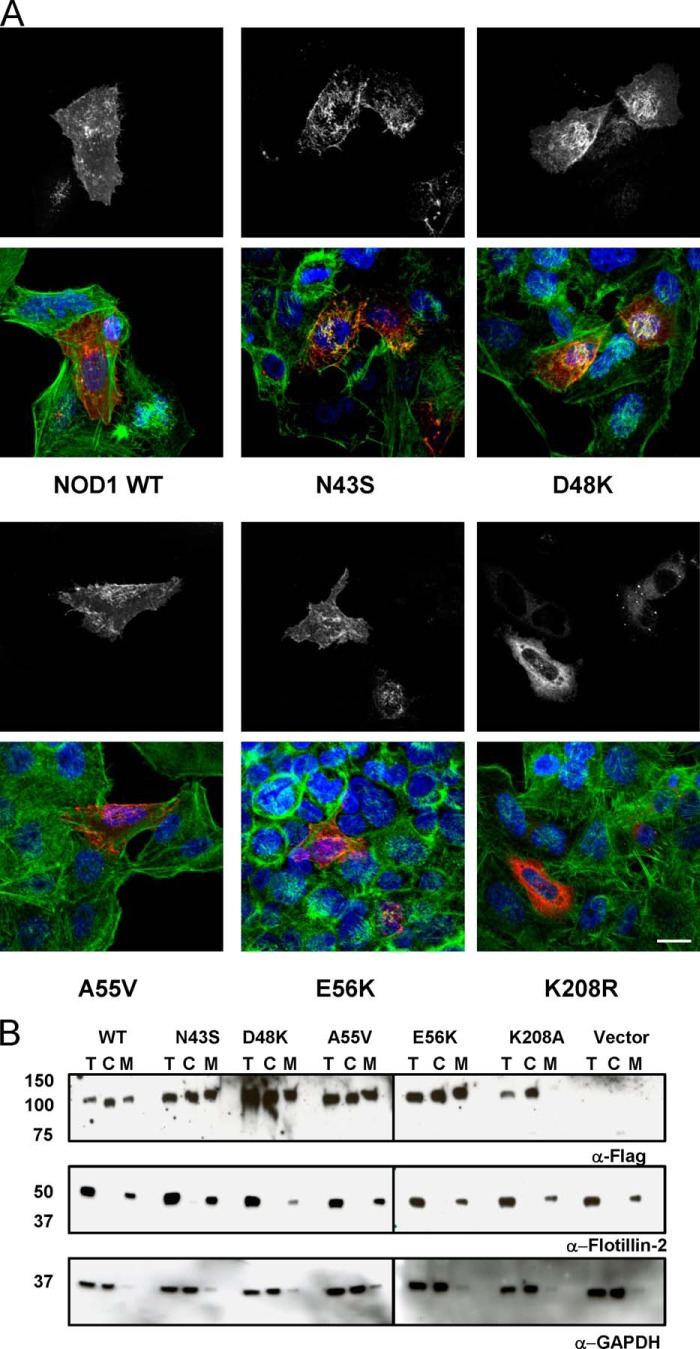
**The cellular localization of NOD1 SNPs is comparable with that of wild-type NOD1.** *A*, immunofluorescence studies of FLAG-tagged NOD1 constructs transiently transfected into HeLa cells using FuGENE 6. NOD1 proteins (*white* in *top panels* and *red* in *bottom panels*) were detected with mouse anti-FLAG M2 and goat anti-mouse Alexa Fluor 546 antibodies. DNA (*blue*) was stained with DAPI, and actin (*green*) was stained with phalloidin-FITC. *B*, subcellular fractionation of FLAG-NOD1 constructs was performed using a Subcellular Fractionation kit. Samples were probed with antibodies against FLAG to detect NOD1, Flotillin-2 to detect the membrane fraction, and GAPDH to detect the cytoplasmic fraction. *T*, total lysate; *C*, cytoplasmic fraction; *M*, membrane fraction. Images are representative of at least two (*A*) or three (*B*) independent experiments. *Scale bar*, 10 μm.

Upon activation, NOD1 and NOD2 undergo partial relocalization to the plasma membrane to interact with RIP2 and mediate signaling (5–8). We therefore checked the cellular distribution of a representative selection of our constructs. Indirect immunofluorescence studies showed the cellular localization of N43S, D48K, A55V, and E56K to be broadly comparable with that of wild-type NOD1. Each protein showed localized patches of membrane-associated protein in addition to a dispersed cytoplasmic staining (Fig. 4*A*). Consistent with previous reports, a Walker A mutation, K208R, caused loss of membrane localization and a strong, diffuse cytoplasmic signal (6, 40) (Fig. 4*A*). Subcellular fractionation comparing the proportion of NOD1 proteins in the cytoplasm and the membrane confirmed this pattern of distribution. As expected, Flotillin-2 and GAPDH were almost exclusively membrane-associated and cytoplasmic, respectively, showing the purity of the subcellular fractions (Fig. 4*B*). Consequently, the impaired NFκB signaling observed with the mutants N43S and E56K in the NOD1 CARD does not result from either a loss of protein expression or protein mislocalization.

##### Mutation of Surface-exposed Residues Does Not Perturb the Secondary Structure of the NOD1 CARD

**FIGURE 5.**
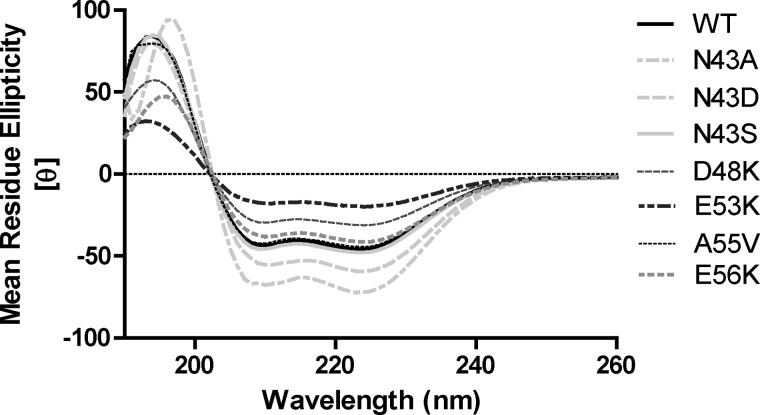
**The secondary structure of NOD1 SNPs is unaltered.** Recombinant NOD1 CARDs at a concentration of 0.4 mg/ml in 25 mm sodium phosphate, pH 7.0, 100 mm sodium fluoride, 1 mm TCEP were analyzed by circular dichroism. The far-UV (190–260 nm) spectra indicate a strong α-helical trace for the wild-type and mutant CARD constructs as labeled with a maximum at 193 nm and minima at 208 and 222 nm. [θ] is the mean residue ellipticity. Plots are representative of at least three independent experiments.

The lack of significant changes in the subcellular localization of the NOD1 mutants described above suggests that these constructs are folding properly. However, it does not provide conclusive proof of correct folding and does not inform on any of the other mutants. To confirm the impact of the point mutants on protein folding, we expressed wild-type and mutant recombinant NOD1 CARD domains (amino acids 15–110) in *Escherichia coli*. Neither the L44A nor D54K construct showed any protein expression, consistent with a critical structural role in the hydrophobic core and in forming internal salt bridges, respectively. All other constructs were expressed and purified in a manner comparable with the wild-type CARD and produced circular dichroism spectra consistent with an α-helical protein (Fig. 5). SELCON3 analysis indicated that all NOD1 CARD proteins were ∼80% helical, 5% turn, and 15% unordered. Hence, although the defect in signaling when mutating Leu-44 or Asp-54 may result from protein misfolding, this is highly unlikely when mutating Asn-43, Glu-53, or Glu-56.

##### Signaling-defective Mutants Can Still Interact with the RIP2 CARD

**FIGURE 6.**
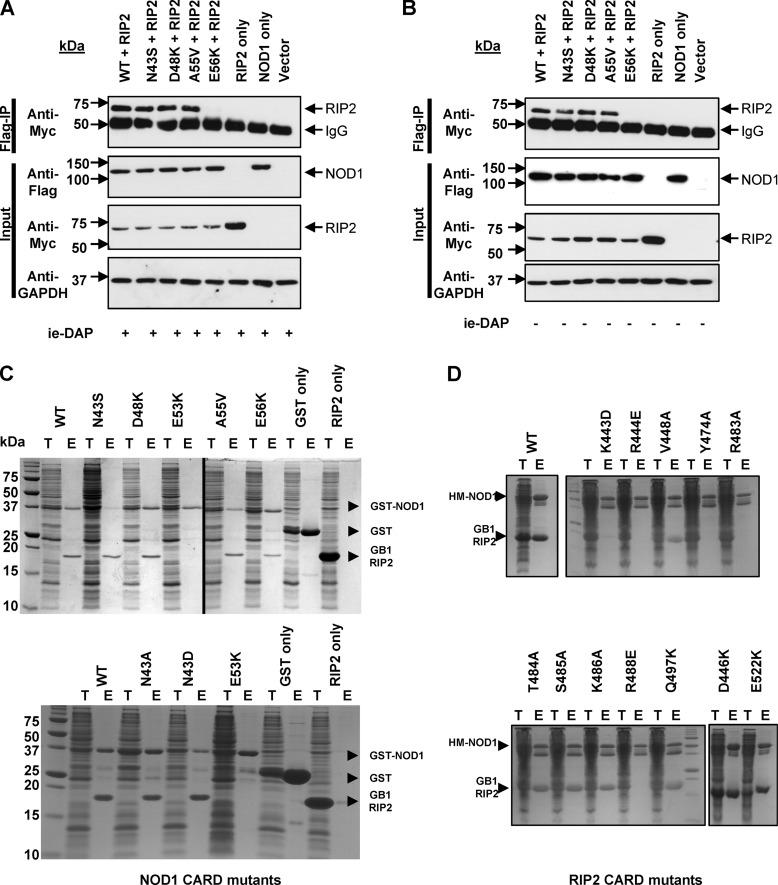
**Asn-43 mutants still interact with RIP2.** Co-immunoprecipitation experiments were performed in HEK293 cells transfected with the constructs detailed in either the presence (*A*) or absence (*B*) of stimulation with 100 ng/ml iE-DAP. Complexes were immunoprecipitated using anti-FLAG antibody immobilized on Protein G-coated Dynabeads and analyzed with the antibodies detailed. Blots are representative of at least three separate experiments. co-purification of *E. coli*-expressed GST-NOD1 CARD and GB1-RIP2-CARD (*C*) or His-MBP-NOD1-CARD and GB1-RIP2-CARD (*D*). Expressed proteins were pooled during lysis, co-purified with glutathione-Sepharose or amylose resin as appropriate, and detected by Instant Blue (Expedeon) staining. In both *C* and *D*, *T* represents total lysate, and *E* represents eluted fraction. The position of recombinant proteins is marked. Images are representative of at least three independent experiments.

Experiments with double and triple mutants in helix 2 have led to the suggestion that helix 2 helps stabilize interaction with RIP2 (18). We hypothesized that the impaired signaling upon mutation of Asn-43 could result from disruption of the interaction with RIP2. To test this, we performed co-immunoprecipitations in HEK293 cells transfected with pCI-RIP2-Myc-His and both wild-type and mutant pCMV-FLAG-NOD1 constructs. As expected given their normal signaling profile, wild-type NOD1, D48K, and the helix 3 polymorphism A55V all immunoprecipitated RIP2. The inactive mutant E56K did not (Fig. 6*A*) (18). However, RIP2 was successfully immunoprecipitated by the signaling-impaired mutant N43S (Fig. 6*A*). The same pattern of immunoprecipitation was seen with (*A*) and without (*B*) stimulation by iE-DAP (Fig. 6). Therefore, the impaired NFκB-mediated signaling of N43S is not the result of a failure to recruit RIP2 to the signaling complex. Together with the results of others (18) this suggests that singular mutations of helix 2 do not disrupt interaction between NOD1 and RIP2 but can, however, interfere with signaling.

Immunoprecipitation studies do not rule out the possible involvement of other bridging proteins in the interaction between NOD1 and RIP2. We returned to recombinant proteins to address this possibility. Although recombinant NOD1 CARD constructs could be readily expressed and purified to homogeneity, we were unable to produce sufficiently stable recombinant RIP2 CARD to facilitate biophysical or structural study of the NOD1-RIP2 interaction. To overcome this problem, we modified the recently reported co-expression system used to study interaction between NOD2 and RIP2 (33). We expressed either GST-NOD1 or His-MBP-NOD1 fusions and GB1-RIP2 fusion CARDs in separate cultures, resuspended the cells in lysis buffer, and pooled the samples prior to sonication. Lysed cells were clarified, and the soluble fraction was mixed with affinity resin to purify the NOD1 CARD. Co-purification of GB1-RIP2 was determined by PAGE. Using this novel co-sonication technique we were able to show that, consistent with the co-immunoprecipitation study, recombinant RIP2 protein was pulled down using wild-type NOD1 CARD and the mutants D48K, N43S, N43A, N43D, and A55V (Fig. 6*C*). As expected, E53K failed to interact with RIP2 as did the negative controls of GST alone and resin alone. Interestingly, E56K, which did not immunoprecipitate RIP2 in HEK293 cells, was able to pull down RIP2 in this recombinant system. This may be due to the higher protein concentrations in the pulldown system or, consistent with our own published data (32), reflect a less critical role for Glu-56 in maintaining interaction with RIP2. Due to the inability of either D54K or L44A to be expressed as recombinant proteins in *E. coli*, we were unable to assess their interaction with RIP2 by this method.

We also used our co-sonication assay to gain further insight into the role of residues on the surface of RIP2 that are important for interaction with NOD1. We prepared a series of mutants in the RIP2 CARD based on their proximity to the core basic residues (Arg-444, Arg-483, and Arg-488) previously identified to be involved in NOD1 interaction (18). Mutation of Val-448, Thr-484, Ser-485, Lys-486, Gln-497, Asp-446, and Glu-522 had no impact on the interaction with NOD1 (Fig. 6*D*). In contrast, in addition to Arg-444, Arg-483, and Arg-488, mutating Lys-443 and Tyr-474 stopped interaction between NOD1 and RIP2 CARDs (Fig. 6*D*).

##### Mutating Asn-43 in Helix 2 Alters the Propensity of the NOD1 CARD to Form Dimers

**FIGURE 7.**
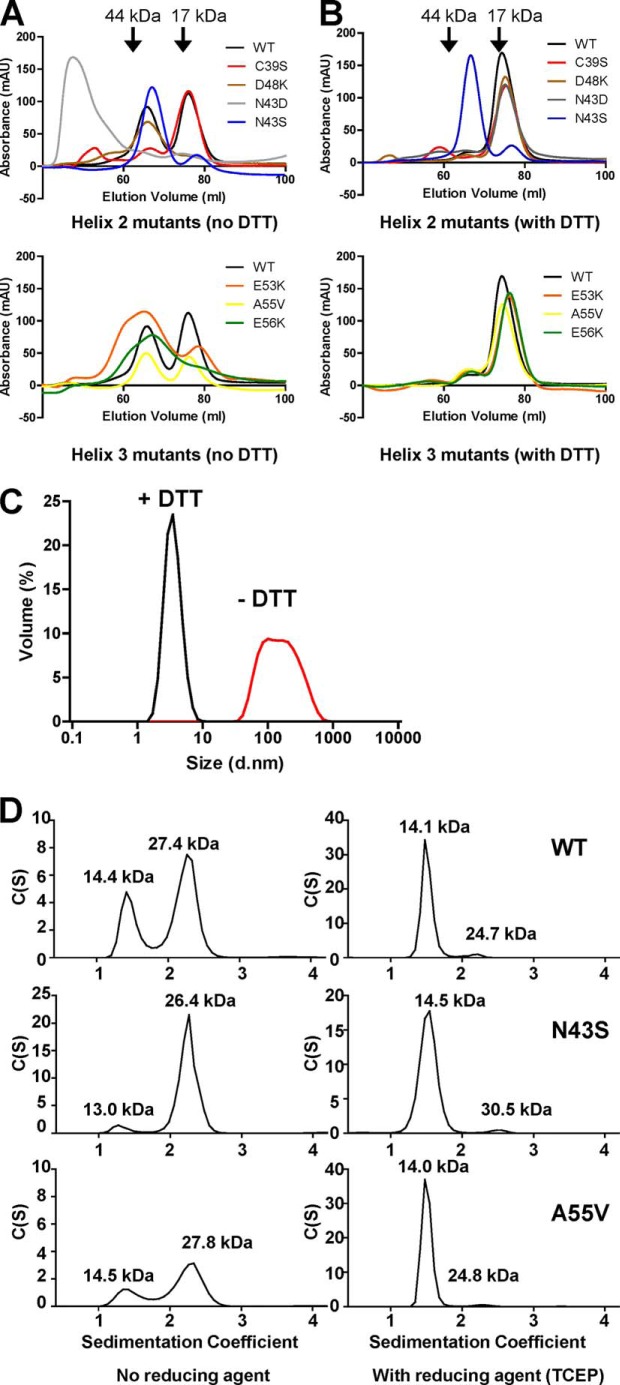
**Mutation of Asn-43 in helix 2 alters the multimerization properties of the NOD1 CARD.** *A* and *B*, size exclusion chromatography of recombinant NOD1 CARD constructs from helix 2 (*top*) and helix 3 (*bottom*) in the absence (*A*) or presence (*B*) of the reducing agent DTT. Constructs are colored as follows: wild-type NOD1, *black*; C39S, *red*; D48K, *brown*; N43D, *gray*; N43S, *blue*; E53K, *orange*; A55V, *yellow*; and E56K, *green. C*, dynamic light scattering confirms the aggregation of N43D in the absence of reducing agent. *Red line*, no reducing agent; *black line*, reducing agent (DTT) present. *D*, sedimentation velocity analytical ultracentrifugation of wild-type, N43S, and A55V recombinant NOD1 CARDs in the absence (*left-hand panels*) and presence (*right-hand panels*) of the reducing agent TCEP. Molecular masses as determined by the use of standards are denoted by *arrowheads* in *A* and *B*. Calculated molecular masses are labeled above the individual peaks in *D*. Data are representative of at least three independent experiments. *mAU*, milliabsorbance units; *d.nm*, diameter in nm.

Residues in helix 2 contribute to the dimer interface in the NOD1 crystal structures (Table 2). This led us to ask whether alterations in dimer formation could contribute to the functional changes we had observed. We used size exclusion chromatography of recombinant NOD1 CARD constructs to determine the oligomeric status of NOD1 helix 2 and helix 3 mutants (Fig. 7, *A* and *B*). In the absence of reducing agent, wild-type NOD1 CARD showed similar proportions of both monomer and dimer (Fig. 7*A*). As expected, replacement of Cys-39 with serine resulted in a loss of the dimeric form. The helix 2 mutants N43S and D48K were both predominantly dimeric, whereas N43D eluted in the void volume due to the formation of large aggregates. Aggregate formation in the absence of reducing agent by N43D was confirmed using dynamic light scattering (Fig. 7*C*). Helix 3 mutants showed evidence of both monomeric and dimeric NOD1 in the absence of reducing agent (Fig. 7*A*, *bottom panel*). In the case of E53K and E56K, the dimeric peak was broad, suggesting a potentially heterogenous population. In the presence of reducing agent, wild-type NOD1 CARD and all of the helix 2 and helix 3 mutants tested with the exception of N43S were almost entirely monomeric (Fig. 7*B*). N43S in contrast maintained the same elution profile as seen under non-reducing conditions, indicating that the protein was almost entirely dimeric.

To further study these observations, we analyzed N43S, wild-type NOD1, and the helix 3 mutant A55V by analytical ultracentrifugation in both the absence and presence of reducing agent. In the presence of reducing agent, wild-type NOD1 CARD, N43S, and A55V were predominantly monomeric (Fig. 7*D*). The reduction in the proportion of dimer for N43S under these conditions compared with size exclusion chromatography likely results from the use of TCEP, a stronger permanent reducing agent, in the analytical ultracentrifugation. Without reducing agent, a considerable proportion of each protein and almost all of N43S existed as a dimer (Fig. 7*D*). Hence, mutation of Asn-43 in helix 2 alters the likelihood of higher order structure formation between NOD1 CARD protomers that in turn could inhibit signaling.

##### The NOD1-RIP2 Interactions Are Consistent with Involvement of Both a Type I and Type III Interface

Death domains interact using specific interfaces (Table 1). Until recently, the only structure of a CARD complex involved a type III interface between the CARDs of Apaf-1 and procaspase-9 (41). However, recent structures have demonstrated that purified CARDs can use all three interaction types to form oligomeric (36) and polymeric (42, 43) complexes.

Helices 1 and 4 of the RIP2 CARD and helices 2 and 3 of the NOD1 CARD are implicated in RIP2-NOD1 interaction, suggesting involvement of a type I interface (18) (Table 1). In light of our observations that mutation of the surface-exposed Asn-43 in helix 2 of the NOD1 CARD still permits interaction with RIP2, we investigated the potential interfaces involved in the NOD1-RIP2 interaction via comparison with solved CARD·CARD structures.

**FIGURE 8.**
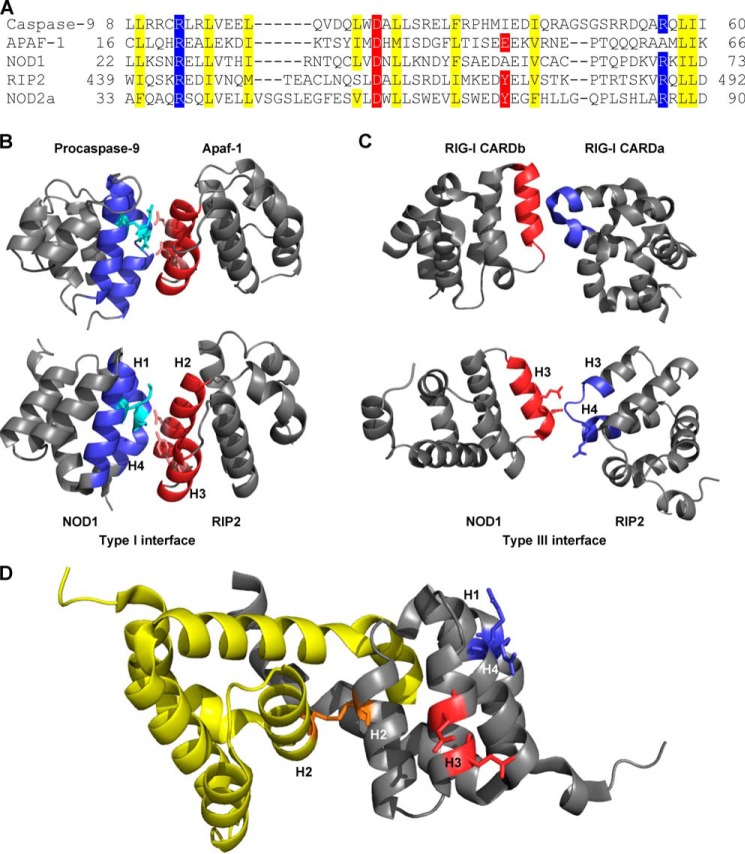
**Involvement of type I and type III interfaces in the interaction between NOD1 CARD and RIP2 CARD.** *A*, structure-based alignment of the regions involved in a type I interface. Type Ia basic residues (*blue*) and type Ib acidic residues (*red*) were identified from the crystal structure of Apaf-1·procaspase-9 CARD complex (Protein Data Bank code 3YGS (41)). Internal hydrophobic residues are highlighted *yellow. B*, the type I interface between procaspase-9 (*top left*) and Apaf-1 (*top right*) was used to align the position of the NOD1 (*bottom left*) and RIP2 (*bottom right*) CARDs, consistent with a type I interaction (*bottom*). The type Ia patch on procaspase-9 and NOD1 and the type Ib patch on Apaf-1 and RIP2 are colored *blue* and *red*, respectively. Side chains of residues reported to be critical for the interaction are shown as *stick* representations. *C*, the type III interface between RIG-I CARDs (Protein Data Bank code 4NQK (36)) (*top*) was used to identify the potential type III interfaces in NOD1 (*bottom left*) and RIP2 (*bottom right*). The acidic type IIIa surface on NOD1 is colored *red*, and the basic type IIIb surface on RIP2 is colored *blue*. Side chains of residues reported to be critical for the interaction are shown as *stick* representations. *D*, the type I (*blue*) and type III (*red*) interfaces of NOD1 remain available for interaction with RIP2 in the helix-swapped NOD1 CARD dimer (Protein Data Bank code 2NSN (39)). The NOD1 protomers are shown in *yellow* and *gray*, and the Cys-39-mediated disulfide bond is shown in *orange*. For clarity, the type I and type III interfaces are only included on one protomer. Structural figures were generated using PyMOL. Key helices on NOD1 and RIP2 are labeled (*H1–H4*).

The type I CARD interaction between Apaf-1 and procaspase-9 uses a type Ia patch centered on two basic residues and a type Ib patch centered on two acidic residues (41). Sequence alignments of the CARDs of Apaf-1, procaspase-9, NOD1, RIP2 and NOD2 CARDa (Fig. 8*A*) indicated that both the NOD1 CARD and NOD2 CARDa contain the same type Ia basic patch as procaspase-9. Mutation of these basic residues by Manon *et al.* (18) (NOD1 Arg-69) and Fridh and Rittinger (33) (NOD2 Arg-38 and Arg-86) abrogated interaction with RIP2. The opposing type Ib patch on RIP2 involves Asp-461 and Tyr-474 (Fig. 8*A*). Mutation of Asp-461 disrupts the interaction of RIP2 with NOD1 or NOD2 (33), although we have shown in this work that Tyr-474 is also necessary for NOD1 interaction. The position of these residues maps well to those in the Apaf-1·procaspase-9 structure (Fig. 8*B*). We can therefore conclude that the NOD1·RIP2 complex involves a type I interaction utilizing a type Ia basic patch in the NOD1 CARD and a type Ib acidic patch on RIP2.

RIP2 also contains a basic type Ia patch consisting of Arg-444 and Arg-488 (Fig. 8*A*) that is essential for the NOD1-RIP2 interaction (18). However, mutation of the NOD1 type Ib surface (Asp-42 (18) and Ala-55 (this work)) (Fig. 8*a*) does not significantly affect signaling (18). As such, the NOD1·RIP2 complex is not likely to include a RIP2 type Ia-NOD1 type Ib interaction. However, the importance of the RIP2 1a interface suggests that the overall complex is likely to involve a RIP2-RIP2 type I interaction.

Glu-53 and Glu-56 from the NOD1 CARD and Arg-483 from the RIP2 CARD are also important for maintaining the interaction between NOD1 and RIP2. We compared the locations of these residues with the interfaces reported in the recent oligomeric RIG-I CARD complex (36) and found that they corresponded to the type III interface (Fig. 8*C*). Specifically, NOD1 residues form part of a type IIIa interface, and the RIP2 residue is part of a type IIIb interface. Together these observations suggest that NOD1 and RIP2 CARDs mediate formation of an oligomeric structure involving both type I and type III interfaces.

Given the increased propensity for N43S to form dimers, we also investigated the availability of the NOD1 type I and type III interfaces in the dimeric form of the NOD1 CARD. Importantly and consistent with the ability of N43S to still bind RIP2, both the type I and type III interface reside on the external surface of the dimer (Fig. 8*D*). Consequently, RIP2 should still be able to bind dimeric NOD1 but may not be capable of forming a higher order oligomeric structure necessary for successful signaling.

## DISCUSSION

Immune signaling pathways require tight regulation and control to ensure an appropriate cellular response to pathogens and cellular danger signals. The NLR proteins NOD1 and NOD2 propagate signaling through engagement of the adaptor protein RIP2. In the work presented here, we focused on the nature of the interaction between the CARDs of NOD1 and RIP2. Using cell-based techniques and recombinant protein analysis we showed that helix 2 of the NOD1 CARD is not part of the interface involved in RIP2 binding. However, mutation of the residue Asn-43 in helix 2 impaired receptor signaling most likely as a result of alterations in the propensity of the NOD1 CARD to self-associate. In addition, we identified two additional residues in RIP2, Lys-443 and Tyr-474, mutation of which abrogated interaction with NOD1.

Disruption of helix 2 in NOD1 affects receptor function but not as a result of inhibiting the interaction with RIP2 (Figs. 3 and 6 and Ref. 18). Helix 2 is also the only region of the NOD1 CARD implicated in both CARD·CARD dimerization and signaling function (Table 2). Although the NOD1 CARD forms a dimer in the crystal structure (38, 39), clear cut evidence for physiological formation of the dimer or a potential role in the activation of signaling through reduction of the Cys-39-mediated disulfide has yet to be demonstrated. However, our observations that N43S had an increased propensity to form dimers and that N43D formed larger aggregates suggest that these processes may be intrinsically linked and could provide a potential mechanism for receptor dysfunction.

More specifically, residues in NOD1 that are important for RIP2 binding remain accessible in the dimeric form of the NOD1 CARD (Figs. 1*B* and 8*D*). These include Arg-69 (type Ia basic interface) and Glu-53 and Glu-56 (type IIIa acidic interface). Residues in NOD1 recently reported to bind ubiquitin (Glu-84 and Tyr-88 (19)) are also accessible in the dimeric form. Consequently, the ability of RIP2 to be recruited to and bind the NOD1 CARD in the monomeric and dimeric forms is to be expected. However, the relative orientation of the RIP2 molecules recruited may differ for the monomeric and the dimeric forms of the NOD1 CARD. Post-translational modification of RIP2 via ubiquitination and/or phosphorylation has been reported to play a crucial role in signal transduction (9, 10). Indeed, autophosphorylation of Tyr-474 in the RIP2 CARD is crucial for the full engagement of signaling following activation of NOD2. It is tempting to speculate that alterations in post-translational modifications of RIP2 resulting from altered protein accessibility could explain the impact of Asn-43 mutation on receptor signaling. This argument is strengthened by the direct involvement of RIP2 Tyr-474 in the interaction with NOD1 (Fig. 6*D*). To fully understand the mechanistic impact of these mutations, a more detailed study investigating post-translational modifications of RIP2 and NOD1 is required.

The alteration in the propensity of the NOD1 CARD to dimerize and/or multimerize following mutation of Asn-43 draws parallels with other recently described immune signaling networks. For instance, studies on the CARMA1/Bcl10/MALT1 signalosome (42) and of RIG-I (36) and MDA-5 (44–46) signaling serve as a reminder of the potential importance of CARD oligomerization in innate immune signaling. These studies coupled with recent work looking at aggregation of apoptosis-related speck-like protein containing a CARD (ASC) raise the possibility that the NOD1 CARD may be functioning as a nucleation point for subsequent assembly of a RIP2 oligomer (47, 48). It should be noted that for an NLR-related family of proteins in plants dimerization of their coiled coil and Toll-interleukin 1 receptor effector domains plays a pivotal role in activation and signaling (49, 50), making it tempting to speculate that regulation of the oligomerization status of NLR effector domains is a conserved feature of NLR signaling.

Currently, we lack high resolution structural information for a complex between an NLR effector domain and its adaptor protein. However, the structures of other death domain fold proteins, such as the PIDDosome (37), show that death domain family members can form multiprotein complexes with each subunit utilizing at least three independent types of interaction (types I, II, and III) (37). A type I interaction, exemplified by the CARDs of Apaf-1 and procaspase-9, contains residues from helices 2 and 3 of one protein (a Ib interface) interacting with residues from helices 1 and 4 from a second protein (a Ia interface). It has been assumed previously that all CARD·CARD complexes would adopt a conformation very similar to that of Apaf-1·procaspase-9. The recent crystal structure of the RIG-I CARD complex makes it clear that this is not the case and that CARD-CARD interactions include multiple interface types (36). Our analysis and that of others (18) highlight the importance of helix 3 in the NOD1 CARD for interaction with RIP2 but also reveal that helix 2 is not directly involved in the interface. By comparing the locations of residues crucial for the NOD1-RIP2 CARD interaction with those involved in the Apaf-1·procaspase-9 and RIG-I structures, we have shown that both type I and III interfaces are likely to be involved in NOD1-RIP2 interface. Coupled with the potential involvement of a type I RIP2-RIP2 interface, this would be consistent with production of a larger oligomeric structure following signal activation. However, confirmation of this will have to wait until we have a molecular structure of the RIP2·NOD1 CARD complex.

The complexity of CARD-mediated signaling has been highlighted by a number of recent studies. Kersse *et al.* (34) have identified multiple interfaces in interactions involving the CARD of procaspase-1. For example, Asp-27 is crucial for interaction with ASC via a type I interface, and Arg-45 is part of a proposed type III interface mediating auto-oligomerization and facilitating interaction with RIP2. Meanwhile, Proell *et al.* (35) have shown that multiple interaction surfaces on the CARD of ASC are crucial for inflammasome formation and interaction with caspase-1. Mutation of two patches, one composed of residues in helices 1, 3, and 4 and the other from residues in helices 2 and 3, resulted in a loss of ASC focus assembly (35). Furthermore, Fridh and Rittinger (33) identified clear differences in the binding surfaces involved in NOD1-RIP2 and NOD2-RIP2 interactions. NOD1 and RIP2 interact via acidic and basic patches on the respective proteins, whereas basic residues on the first CARD of NOD2 interact with acidic residues on RIP2. They also identified acidic residues in RIP2 (Asp-461, Glu-472, Glu-475, and Asp-492) that contribute to the interaction with NOD1, suggesting either a larger interaction surface composed of acidic and basic patches or an alternative interface conformation. We identified two further RIP2 residues involved in NOD1 binding. The first of these, Lys-443, fits firmly with the regions proposed by Manon *et al.* (18). The second, Tyr-474, is located in the group of residues identified by Fridh and Rittinger (33) and is autophosphorylated following RIP2 activation by NOD2 (9). Mutation of Tyr-474 to Phe reduced but did not stop the interaction between NOD2 and RIP2 (9). Together these observations suggest that Tyr-474 and surrounding residues may serve important functional roles in signal transduction for both NOD1 and NOD2 pathways. Our observations help to confirm the widening view that CARD-CARD interactions are indeed complex and highlight the need for further studies to address not just the structural basis of complexes, such as that formed by NOD1 and RIP2, but also the precise nature and regulation of the post-translational modifications involved in signal transduction.

## Footnotes

3 The abbreviations used are:

NLR: nucleotide-binding leucine-rich repeat-containing
Apaf-1: apoptosis protease-activating factor 1
CARD: caspase recruitment domain
iE-DAP: d-Glu-*meso*-diaminopimelic acid
NOD1/2: nucleotide oligomerization domain-containing 1/2
RIP2: receptor-interacting protein 2
MBP: maltose-binding protein
TCEP: tris(2-carboxyethyl) phosphine
ASC: apoptosis-related speck-like protein containing a CARD
GST: glutathione-*S*-transferase
PIDD: p53-induced death domain protein.
